# Molecular evidence for horizontal transmission of chelonid alphaherpesvirus 5 at green turtle (*Chelonia mydas*) foraging grounds in Queensland, Australia

**DOI:** 10.1371/journal.pone.0227268

**Published:** 2020-01-09

**Authors:** K. Jones, G. Burgess, A. M. Budd, R. Huerlimann, N. Mashkour, E. Ariel

**Affiliations:** 1 College of Public Health, Medical and Veterinary Sciences, James Cook University, Townsville, Queensland, Australia; 2 Centre for Sustainable Tropical Fisheries and Aquaculture, James Cook University, Townsville, Queensland, Australia; 3 Centre for Tropical Water and Aquatic Ecosystem Research, James Cook University, Townsville, Queensland, Australia; University of Melbourne, AUSTRALIA

## Abstract

Fibropapillomatosis (FP) is a marine turtle disease recognised by benign tumours on the skin, eyes, shell, oral cavity and/or viscera. Despite being a globally distributed disease that affects an endangered species, research on FP and its likely causative agent chelonid alphaherpesvirus 5 (ChHV5) in Australia is limited. Here we present improved molecular assays developed for detection of ChHV5, in combination with a robust molecular and phylogenetic analysis of ChHV5 variants. This approach utilised a multi-gene assay to detect ChHV5 in all FP tumors sampled from 62 marine turtles found at six foraging grounds along the Great Barrier Reef. Six distinct variants of ChHV5 were identified and the distribution of these variants was associated with host foraging ground. Conversely, no association between host genetic origin and ChHV5 viral variant was found. Together this evidence supports the hypothesis that marine turtles undergo horizontal transmission of ChHV5 at foraging grounds and are unlikely to be contracting the disease at rookeries, either during mating or vertically from parent to offspring.

## Introduction

Fibropapillomatosis (FP) is a marine turtle disease, characterised by the growth of benign tumours on the skin, eyes, shell, oral cavity and/or viscera. This disease has been reported in every species of marine turtle but predominantly affects the endangered green turtle (*Chelonia mydas*) [1]. Although benign, FP tumours are physically debilitating as their positioning can impair vision, feeding and locomotion [2–4], leaving the affected turtle with increased vulnerability to predation, starvation and boat-strike. Turtles with FP are typically chronically stressed [5] and immunosuppressed [5, 6] and are therefore susceptible to secondary infections and opportunistic pathogens. FP has a global distribution, with prevalence rates varying spatially and temporally [1]. Such variance in disease prevalence creates a unique challenge for environmental managers; a more solid understanding of this disease is critical for the development of informed management plans.

Although the causative agent of FP is yet to be confirmed, studies have consistently reported a link between FP tumours and the presence of a herpesvirus [7–9]. As this virus could not be cultured in vitro until recently [10], there has been an increase in studies utilizing molecular methods to better understand this herpesvirus [11–23]. These studies have added to the body of evidence linking a turtle-specific herpesvirus, known as chelonid alphaherpesvirus 5 (ChHV5), and FP. As such, ChHV5 is now generally accepted as the likely causative agent of this disease.

Genetic variation of ChHV5 is an emerging field, wherein four distinct clades of ChHV5 have been described globally (eastern Pacific, western Atlantic and eastern Caribbean, Midwest Pacific, and Atlantic [24]. Samples collected from turtles from a particular region tend to cluster into the associated phylogeographic group. For example, samples from Brazil cluster into the Atlantic [23] and samples from Ecuador into Pacific [25]. Variation in ChHV5 has also been described at more local levels, with four variants in Florida [26] and six variants in Brazil [23]. A geographic influence on the distribution of these variants has been reported in both Brazil [23], Florida [26], Hawaii [27] and most recently, Australia [28]. Characteristically, turtles at a particular foraging ground are infected with the same viral variant, which is distinct from variants found at other foraging locations within a particular region [23, 24, 26–29].

Marine turtles have a complex life-history, spanning multiple habitats, which makes it difficult to pinpoint the stage and location that ChHV5 transmission occurs. Hatchlings emerge from rookeries in tropical and subtropical regions where they then undertake a pelagic existence. Several years later, they recruit into inshore foraging grounds as juveniles [30]. The turtles at these foraging grounds originate from multiple regional rookeries [31–33] and have strong site fidelity to both the foraging ground they inhabit and the rookery from which they originated. Turtles will attempt to return to their rookery to breed and nest at the onset of sexual maturity [34]. Due to this natal philopatry, turtles originating from rookeries in a particular region are genetically distinct stocks. Transmission of ChHV5 may occur at the rookery, the foraging ground, or in transit between these habitats. Assessing distribution patterns of the virus may provide an indication as to which of these locations, if any, is the site of transmission.

If ChHV5 transmission is vertical, occurring at rookeries from parent to offspring, a homogeneous distribution of genetic variance of ChHV5 at each rookery would be expected [26]. In such a situation, a link between viral variant and turtle origin (genetic stock) would also be expected, regardless of sampling location. Conversely, if ChHV5 transmission is occurring horizontally at foraging grounds, a homogeneous distribution of genetic variance of ChHV5 at each foraging ground (and heterogeneous distribution over multiple foraging grounds) would be observed [26]. In this case, a link between viral variant and host origin would be less likely. As FP predominantly affects juvenile/immature green turtles, horizontal transmission between adult females at rookeries is less likely [1]. Moreover, the heterogeneity in viral variant distribution observed in previous studies, coupled with high FP prevalence in juvenile/immature turtles [1], has led to the hypothesis that ChHV5 transmission occurs upon recruitment into inshore foraging grounds after the pelagic phase in the marine turtle life-cycle [1, 24, 26]. Whilst this hypothesis is widely accepted, a molecular link between viral variant and host origin has never been investigated using molecular methods.

Although a global understanding of FP and ChHV5 is emerging, Australia is an understudied region. The Great Barrier Reef (GBR) supports some of the largest green turtle rookeries and foraging populations in the world [35, 36] and relies heavily on the presence of green turtles for ecotourism [37, 38]. Turtles with FP have been observed at multiple locations on the GBR since the 1970’s [39] yet, to date, only two molecular studies on ChHV5 have generated and analyzed sequence data from samples collected in Australia [16, 28]. A geographic influence on viral variant distribution along the north Queensland coast was recently reported [28], but a link between viral variant and host origin was not assessed. Moreover, the presence and distribution of ChHV5 along the entire coast of the GBR has not been investigated and a solid understanding of FP and ChHV5 on the GBR is yet to be established. As a result, marine turtle management plans are unable to detail an effective means of managing this threat.

In order to inform management decisions and improve conservation outcomes for *C*. *mydas* and other vulnerable turtle species, this study aims to improve our understanding of ChHV5 along the GBR through the following objectives: Firstly, this study will improve the resolution of the current phylogeny of ChHV5 in Australia by generating a more robust sequence data set than has previously been used, including a larger sample size and increased geographical locations. Secondly, the relationship between host genetic stock and viral variant will be assessed in order to clarify the mechanisms of viral transmission.

## Materials and methods

### Sample origin

**Fig 1:**
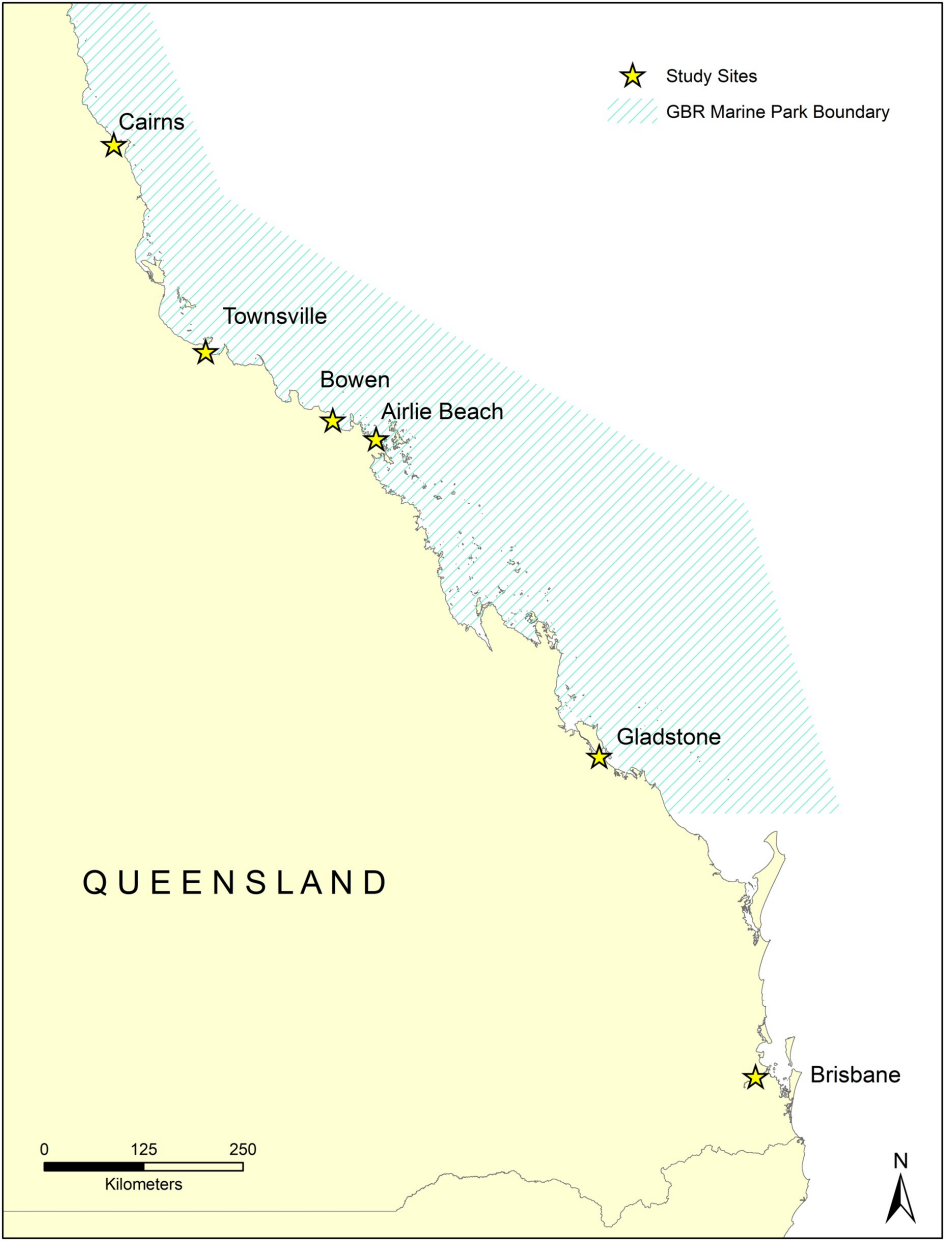
Samples (n = 62) were collected from six locations along the Queensland coast of Australia; Brisbane (n = 7), Gladstone (n = 4), Airlie Beach (n = 1), Bowen (n = 27), Townsville (n = 22), and Cairns (n = 1). Five of these sites are located within the Great Barrier Reef (GBR) Marine Park, whilst Brisbane is located just south of the GBR boundary (indicated by hatched area).

A total of 59 green turtles, two loggerhead (*Caretta caretta*) turtle and one green/hawksbill (*Eretmochelys imbricata*) hybrid turtle were sampled across five locations along the GBR. The majority of samples used in this study were collected opportunistically from turtles with FP tumours, captured using the rodeo capture technique [40] at various foraging grounds along the GBR (Fig 1). The remaining tumour samples were collected during necropsy and others were donated (see S1 Table). The final dataset consisted of turtles from waters near Brisbane (n = 7), Gladstone (n = 4), Airlie Beach (n = 1), Bowen (n = 27), Townsville (n = 22), and Cairns (n = 1). These turtles were predominantly juveniles, with an age class breakdown for the green turtles of 53 juveniles, five sub-adults and one adult. Both loggerheads were immature [41]. The green/hawksbill hybrid (QA47488) was believed to be immature, based on ranges for both hawksbill [42] and green turtles [43].

All live turtles were sampled under permits from James Cook University Animal Ethics Committee (A1501 and A1971), Department of Environment and Science (WISP06619309 and WISP13754613) and Great Barrier Reef Marine Park Authority (G10/33220.1 and G36593.1).

### Sample collection

All live turtles were flipper-tagged with a unique alpha-numeric inscribed titanium tag (Stockbrands Company, Pty. Ltd., Perth, Western Australia), and had their curved carapace length (CCL ± 2 mm) measured using a flexible tape measure. Tumour samples were collected with a paired skin sample from the trailing edge of the front flipper of each turtle. Tissue samples were collected using fresh, sterile, disposable scalpel blades and stored in cryovials containing 90% ethanol. Samples were stored at 4°C prior to DNA extraction.

### DNA extraction, primer design and Polymerase Chain Reaction (PCR)

DNA was extracted using the Promega Wizard^®^ SV Genomic DNA Purification System according to the manufacturer’s instructions with the exception of an additional 10μL of proteinase K used per reaction. Final DNA concentration was obtained by spectrophotometric analysis (Implen Nanophotometer), using the ratios of absorption at 260nm versus 280nm to determine DNA purity.

Primers were designed to target the full-length sequence of three genes within the ChHV5 genome; 1) glycoprotein B (gB), 2) sialyltransferase (F-sial) and 3) DNA polymerase (DNApol). The DNApol gene has been used extensively to determine the presence or absence of ChHV5 [12, 13, 15, 17, 20, 21, 23, 24, 26, 29, 44] due to the highly conserved nature of the gene [45, 46]. Conversely, the gB gene codes for glycoproteins which are located on the surface of the virion and therefore in contact with the host immune system, likely increasing selection pressure. This antigenic nature of gB has led to sequence variability, making it an ideal candidate gene for phylogenetic studies [46–48]. Moreover, [28] demonstrated that this gene is effective in determining ChHV5 phylogeny in Australia. The F-sial gene is atypical of herpesviruses and poorly understood, but has been suggested to play a role in ChHV5 pathogenesis [49].

We also designed and optimised a set of four overlapping primers pairs for gB. Although each of these overlapping primer pairs could be used individually to detect and sequence fragments of ChHV5, it was considered as one assay for the purpose of this study (referred to herein as gB FullOverlap 1–4). All primer sets were designed to include the start and stop codons within the resulting amplicon; primers targeting these regions were placed outside of the target genes so that the resulting sequences could be trimmed to the open reading frame (ORF). The gB primer pairs outside the ORF were designed using an alignment of two ChHV5 gB sequences available from GenBank (National Center for Biotechnology Information; NCBI, Bethesda, Maryland), while primers pairs within the ORF were designed from an alignment of 17 ChHV5 gB sequences. The F-Sial and DNApol primer sets were similarly designed from an alignment of two ChHV5 F-Sial sequences and two ChHV5 DNApol sequences respectively. All primers were designed using AlleleID version 7.7 (Premier Biosoft International, Palo Alto, California) and optimised in conventional PCR using a gradient of 50–60°C (Table 1).

**Table 1:** Primer sequences used to target ChHV5 genes of interest (glycoprotein B; gB, sialyltransferase; F-sial and DNA polymerase; DNApol) and a green turtle (*C*. *mydas*) mtDNA gene (D-loop). F = forward, R = reverse.

Primers	Sequence (5′ → 3′)	Amplicon Length (bp)	Target gene	Reference
gB-2873	F: AGTGTCCCTTGGTAGTTG	2873	Complete gB	This study
	R: GCAATAACGAAATCATAAAGTGTA			
gB-Part1-752	F: AGGAGAATCTTTGGTGGC	752	Partial gB	This study
	R: AAGTCGTAAGGATAAGGAGATTT			
gB-Part2 780	F: AATGGGTGTGGGAAAGAG	780	Partial gB	This study
	R: CCGAGTTAATGTGTTGCC			
gB-Part3 855	F: CGCTGCGGGTAGTGAATT	855	Partial gB	This study
	R: CAACGATCCCATTGAGCA			
gB-Part4 786	F: AACTGGTCAACGATCTGAA	786	Partial gB	This study
	R: GGCTCGAATGCAATAACG			
F-Sial-1104	F: AAAAGATGTACTTGGTATTTGTGT	1104	Complete F-Sial	This study
	R: GCTAATGACGTTACGACTTTT			
DNApol-3670	F: AAAACTCGCAAAGAAAAGTATC	3670	Complete DNApol	This study
	R: ATAAGCGGTTTGTCATCAG			
ChM-Dloop-960	F: AACTATAACCTTCCTAGA	960	mtDNA d-loop control region	[50]
	R: TGTAAGTATCCTATTGATT			

PCRs for the F-Sial-1104 and gB FullOverlap 1–4 primer sets were carried out in 20μL reactions consisting of 10μL GoTaq^®^ Green Hot Start Master Mix (Promega), 0.8μM of each primer, ~80ng of template DNA and nuclease-free water to 20μL. PCRs for the gB-Full-2873 and DNApol-3670 primer sets had the same component volumes but utilised GoTaq^®^ Long PCR Master Mix (Promega) due to the target amplicon length. The thermocycling conditions for all primer sets are outlined in Table 2.

**Table 2:** PCR thermocycling protocols for the newly described primers used in this study.

		Primer Set							
		gB FullOverlap 1–4		F-Sial-1104		gB-2873		DNApol-3670	
Step	Cycles	Temperature	Time	Temperature	Time	Temperature	Time	Temperature	Time
**Initial denaturation**	1	95°C	2 min	95°C	2 min	94°C	2 min	94°C	2 min
**Denaturation**	35	95°C	10 s	95°C	10 s	94°C	30 s	94°C	30 s
**Annealing**		60°C	15 s	59°C	15 s	60°C	30 s	60°C	30 s
**Extension**		72°C	30 s	72°C	30 s	72°C	3 min	72°C	4 min
**Final Extension**	1	72°C.	5 min	72°C.	5 min	72°C.	10 min	72°C	10 min

In order to identify the genetic origin of the host turtle, all tumour samples collected from green turtles were also used in a PCR to amplify a 960bp fragment of the mtDNA d-loop control region using the ChM-Dloop-960 primers and associated conventional PCR protocol described in [50].

PCR products were visualised on a 1.2% (w/v) agarose gel and sent to Macrogen (Macrogen Inc., Seoul, Korea) for purification and bi-directional sequencing.

The gB Overlap 1–4, F-sial-1104 and ChM-Dloop-960 raw sequences were imported into Geneious v7.1.5 [51] and assembled for each individual using reference sequences: F-UL27 of HQ878327, F-Sial of HQ878327 and the CmP47.1 haplotype (KF311753.1) respectively. These sequences were then edited where appropriate and trimmed to the ORF. The resulting consensus sequence was then extracted and confirmed to be the correct target using the database of the Basic Local Alignment Search Tool (BLAST) (https://blast.ncbi.nlm.nih.gov/Blast.cgi). In order to avoid sequencing error, any sequence which appeared to be unique to sequences both in the published literature and within our dataset were re-amplified and re-sequenced to a total of three replicates.

Each ChM-Dloop-960 sequence generated here was also compared with known green turtle haplotypes [32, 50, 52] in order to determine the haplotypes of the individual turtles used in this study. Green turtle haplotype frequencies at rookeries around the world form the basis for estimates of which genetic stock a particular haplotype belongs to [32, 50, 52]. In this study, the haplotype of individual turtles was used to provide an indication of the genetic origin of the host turtle. Once identified, the assigned haplotype was then included in the sequence description for both the gB and F-sial sequences.

### Phylogenetic analysis

For gB, a total of 79 sequences including 58 which were generated in this study, were aligned using ClustalW [53] in Geneious v7.1.5 [51]. Only full-length sequences were used, so the final dataset consisted of 2565 positions. This dataset was then imported into Molecular Evolutionary Genetics Analysis Version X (MEGAX; [54]) for evolutionary analysis. Following a model test, the evolutionary history was inferred by using the Maximum Likelihood method based on the Kimura 2-parameter model [55]. Initial trees for the heuristic search were obtained automatically by applying Neighbor-Join and BioNJ algorithms to a matrix of pairwise distances estimated using the Maximum Composite Likelihood (MCL) approach, and then selecting the topology with superior log likelihood value. The rate variation among sites was modelled with a gamma distribution. The tree was drawn to scale, with branch lengths measured in the number of substitutions per site. The analysis involved 79 nucleotide sequences. There were a total of 2565 positions in the final dataset, and all sites were used.

Upon characterisation of the Australian ChHV5 variants, a single sequence which represented each variant was extracted. These representative sequences were aligned with the 21 available reference sequences used for the previous tree using ClustalW [53] in Geneious v7.1.5 [51], resulting in a final dataset of 29 distinct nucleotide sequences and 2565 positions. A simplified phylogenetic tree was constructed to show the position of these variants relative to the available reference sequences. This tree was constructed as above.

For F-sial, 58 sequences generated from this study and ten reference sequences were aligned using ClustalW [53] in Geneious v7.1.5 [51]. The analysis was therefore comprised of a total of 68 nucleotide sequences. Only full-length sequences were used, so the final dataset consisted of 963 positions. This dataset was then imported into MEGAX [54]. Following a model test, the evolutionary history was inferred by using the Maximum Likelihood method based on the Jukes-Cantor model [56].

### Statistical analysis

A Chi-square test was conducted to assess whether there was a relationship between viral variant and host origin. We also assessed whether there was a relationship between viral variant and sampling location. However, due to small sample numbers in some categories, the assumptions of the chi-square test were not met and as such we report values from the Fisher’s exact test.

## Results

All green turtle samples amplified in the Dloop-960 assay whilst the loggerhead and hybrid (green turtle/hawksbill) samples did not. This assay is specifically designed to target green turtle mtDNA, indicating that the hybrid turtle was likely maternally hawksbill. Analysis of sequence data generated from 59 samples from individual turtles that reacted in this assay revealed that most (74.6%) belong to the CmP47.1 haplotype (Table 3). This is the most common haplotype found on the GBR, typically found in rookeries in the Coral Sea, southern GBR and New Caledonia [32, 52]. The remaining 13.6% of individuals were found to belong to CmP80.1 which is also found in the same regions as CmP47.1. Other turtles were found to be haplotypes typically found to originate from the northern GBR (nGBR) region (CmP98.1, 1.7%) and New Caledonia (CmP85.1, 3.4%; CmP44.2, 1.7%). CmP44.1, a haplotype found in both the nGBR and New Caledonia regions, was found in one individual (1.7%). A haplotype known to originate in the Borneo/Sulu Sea region was found in one individual (CmP57.1, 1.7%) whilst another was found to be CmP34.1 (1.7%), a haplotype of as yet unknown origins. The geographic distribution of these haplotypes among study sites varied, with multiple haplotypes identified at each study site where more than one turtle was sampled (Table 3). This distribution and haplotype frequency is consistent with previous reports [50, 52]. These results were included in the sequence descriptions of the relevant turtles for all other sequences generated in this study.

**Table 3:** Summary of haplotype distribution in green turtles in the present study, including the capture location and regions of origin as determined by sequence analysis of the products of the ChM-Dloop-960 assay.

Haplotype	n	Percentage	Region/s of Origin	Observed locations in this study
CmP47.1	44	74.6	Coral Sea	Townsville
			Southern GBR	Bowen
			New Caledonia	Airlie Beach
				Gladstone
				Brisbane
CmP80.1	8	13.6	Coral Sea	Townsville
			Southern GBR	Bowen
			New Caledonia	Brisbane
CmP98.1	1	1.7	Northern GBR	Cairns
CmP85.1	2	3.4	New Caledonia	Bowen
				Gladstone
CmP44.2	1	1.7	New Caledonia	Townsville
CmP44.1	1	1.7	Northern GBR	Bowen
			New Caledonia	
CmP57.1	1	1.7	Borneo	Townsville
			Sulu Sea	
CmP34.1	1	1.7	Unknown (Orphan haplotype)	Townsville

All samples of DNA extracted from FP tumour samples amplified in at least one of the assays, confirming the presence of ChHV5 in all 62 samples (Table 4). None of the paired skin samples amplified in any ChHV5 assay, with the exception of that from turtle QA42923. Of the 62 tumour samples tested, 58 samples reacted in the gB Overlapping 1–4 assay, the FSial-1104 assay and DNApol-3670 assay (Table 4). ChHV5 DNA was detected in 93.5% of samples in each assay, and in 100% of samples overall.

**Table 4:** Number of positive detections of three ChHV5 target genes in FP tumour samples using Polymerase Chain Reaction (PCR).

		Target Genes		
Location	n	DNA Polymerase	Glycoprotein B	Sialyltransferase
Cairns	1	1	1	1
Townsville	22	21	20	20
Bowen	27	25	27	26
Airlie Beach	1	1	1	1
Gladstone	4	3	2	3
Brisbane	7	7	7	7
Total	62	58	58	58

### Phylogenetic analysis

#### Glycoprotein B (gB)

**Fig 2:**
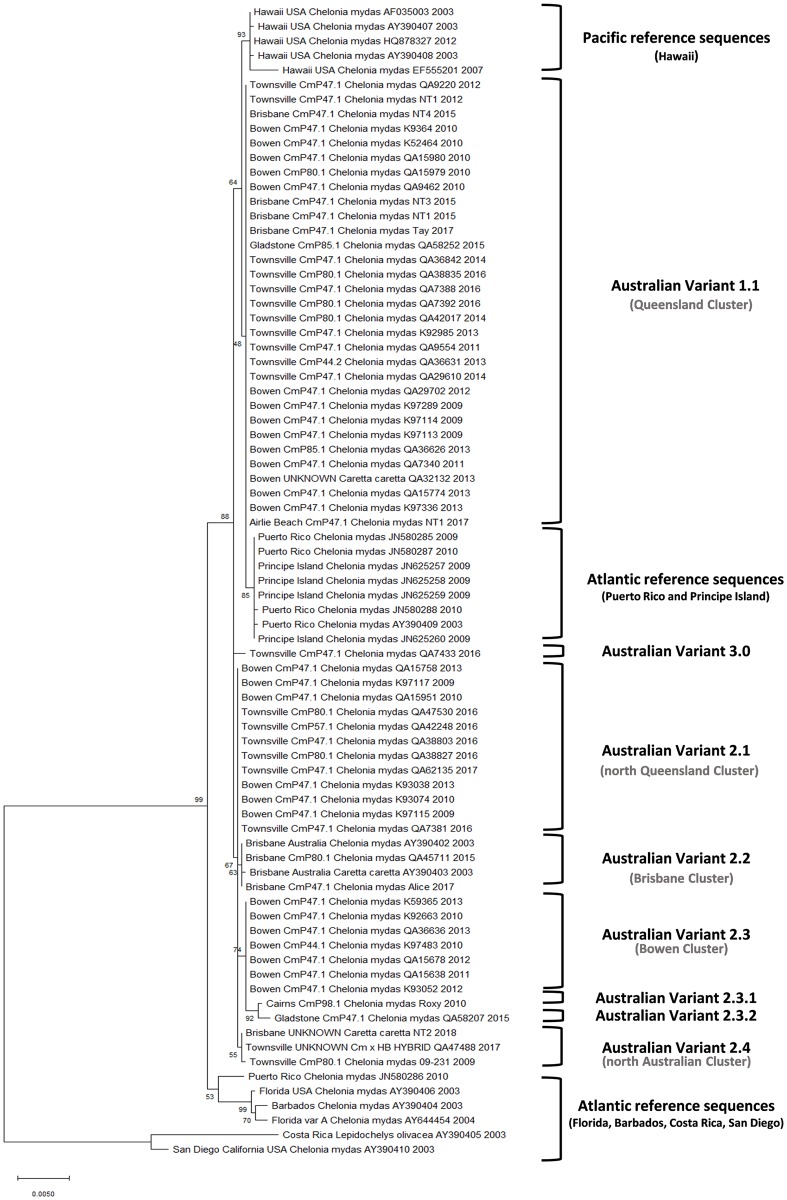
Phylogenetic tree using the Maximum Likelihood method generated from the aligned 2565bp ChHV5 glycoprotein B (gB) gene. The analysis involved 79 nucleotide sequences. Bootstrap values are indicated as a number on each branch and were calculated from 1000 replicates. Individual samples are identified with source location, haplotype, scientific name, tag number, and sample collection year. Sequences retrieved from the GenBank database originating from the Pacific and Altantic are named in the same way, with the accession number in the place of the tag number, and no haplotype information included as it was unknown.

From the nucleotide and phylogenetic analysis of the 58 sequences from this study and 21 available sequences from the NCBI database we show that Australian ChHV5 grouped into four main clusters: a Queensland cluster, north Queensland cluster, Bowen cluster and Brisbane cluster (Fig 2). Both the Queensland and north Queensland clusters have been previously reported [28] whilst the Bowen and Brisbane clusters are newly identified in this study. These results highlight a strong geographic link to viral variant distribution along the Queensland coast, and statistical analysis confirmed that the relationship is statistically significant (X^2^ = 25.016, df = 15, p = 0.011).

The results of this study have also allowed us to better characterize the variants of ChHV5 present in Australia into six clear variants, with subdivisions based on nucleotide differences from the characterised variants. These variants have been named numerically in a hierarchical form, based on whether the variants are first, second or third order clades; second order clades were named to one decimal point (e.g. Australian Variant 2.3) and third order clades were named to three decimal places (e.g. Australian Variant 2.3.1).

Australian Variant 1.1 (Accession number: MK579192) is the most common variant of ChHV5 in Australia, found in turtles along the expanse of the Queensland coast. This variant was found at almost all study sites in the present study, which is consistent with previous descriptions [28]. Here, 53.4% of all turtles whose tumour samples reacted in the gB Overlap 1–4 assay (n = 31) were found to be infected with this variant (Fig 2). This variant is distinct and conserved, with all 31 samples clustering in this clade sharing 100% identity. A single sequence representing Australian Variant 1.1 was generated for further analysis.

Australian Variant 2.1 (Accession number: MK579193) is found only in turtles from the North Queensland region (sites Townsville and Bowen) and is therefore also consistent with previous descriptions [28]. In this study, we report 12 turtles infected with this variant of ChHV5. All Variant 2.1 sequences share 100% similarity, indicating that this variant is also highly conserved. A single sequence representing Australian Variant 2.1 was generated for further analysis.

Australian Variant 2.2 (Accession number: MK579194) is found only in Brisbane as yet, and shares 100% similarity with the Australian green turtle reference sequence (AY390402) (Fig 2). This variant differs from the loggerhead reference sequence (AY390403) by only one nucleotide. However, this is a non-synonymous substitution which alters the amino acid sequence of the resulting protein. Both of these reference sequences (AY390402 and AY390403) were generated from tumour samples from turtles in Moreton Bay (Brisbane), which is consistent with our results. A single sequence representing Australian Variant 2.2 (excluding AY390403) was generated for further analysis.

Australian Variant 2.3 (Accession number: MK579195) is found exclusively in turtles from Bowen (n = 7) and is highly conserved; all sequences in this sub-clade share 100% similarity (Fig 2). A single sequence representing Australian Variant 2.3 was generated for further analysis. Two distinct sequences (Variant 2.3.1 and Variant 2.3.2; accession numbers MK579198 and MK579199 respectively) comprise a subgroup which diverged from Variant 2.3. These sequences share a high similarity with Variant 2.3 (99.8%) yet are unique; Variant 2.3.1 and Variant 2.3.2 differ from Variant 2.3 by 0.2%, but also from each other by 0.2%. Moreover, some of these nucleotide substitutions are non-synonymous, resulting in one amino acid change in Variant 2.3.1 and four amino acid changes in Variant 2.3.2.

Australian Variant 2.4 (Accession number: MK579196) is found in turtles from both Townsville and Brisbane. However, this variant was previously reported as the northern Australian variant, having been found in turtles from Townsville, Cairns and Western Australia [28]. Within this group, two of the sequences (Townsville QA47488 and Brisbane NT2) were identical while the one obtained from Townsville (09–231) differed by one nucleotide. This change, however, was synonymous and therefore the consensus sequence of this variant which was generated for further analysis is an accurate representative of this variant. Interestingly, this variant has a six base pair (bp) deletion that it shares with strains reported from Hawaii and was the most similar to Hawaiian sequences in the alignment. However, this similarity is not reflected in Fig 2, which suggests this variant is most closely related to Variant 2.1.

Australian Variant 3.0 (Accession number: MK579197) is a clear outlier, distinct from all other samples analyzed in this study. Only one turtle from Townsville (QA7433) was infected with this viral variant, which has not been reported prior to this study. Of the Australian variants, this variant shares the highest similarity with Australian Variant 2.1 (99.8% identity) with all nucleotide substitutions being synonymous.

The frequency distribution of the ChHV5 variants among study sites in this study (Table 5) indicates that there is a strong link between viral variant and foraging ground, but that viral distribution within a foraging ground is not strictly homogenous.

**Table 5:** Distribution of chelonid alphaherpesvirus 5 (ChHV5) variants among marine turtles with fibropapillomatosis from six inshore areas in Queensland, Australia.

	Variant							
Location	1.1	2.1	2.2	2.3	2.3.1	2.3.2	2.4	3.0
Cairns	-	-	-	-	1	-	-	-
Townsville	11	6	-	-	-	-	2	1
Bowen	14	6	-	7	-	-	-	-
Airlie Beach	1	-	-	-	-	-	-	-
Gladstone	1	-	-	-	-	1	-	-
Brisbane	4	-	2	-	-	-	1	-
**Total**	31	12	2	7	1	1	3	1

We compared the representative sequences of these variants with a Hawaiian reference sequence (HQ878327) as it is both a well described [49] and the most geographically close to the GBR that is currently available. Whilst all Australian variants shared a high similarity with HQ878327 (Table 6), Variant 2.4 was the most similar as it shared 99.8% identity. It is interesting to note that this shared identity included a six bp which was not observed in any other Australian variants. This deletion appears to be uniquely Hawaiian, as it has not yet been observed in any other location. This deletion also accounted for a consistently observed difference between the Australian variants and HQ878327; all Australian variants, compared to the Hawaiian sequences, had six additional nucleotides resulting in two supplementary amino acids in the protein sequence.

**Table 6:** Summary of variants observed in this study, including number of turtles infected with a particular chelonid alphaherpesvirus 5 (ChHV5) variant (n) and the defining characteristics of these variants. All differences and identity percentages are calculated relative to the full-length glycoprotein B reference sequence available from Hawaii (HQ878327).

Variant	n	Nucleotide Substitutions	Identity (%)	Non-synonymous substitutions
**Variant 1.1**	31	9	99.6	2
**Variant 2.1**	12	11	99.6	2
**Variant 2.2**	2	12	99.5	2
**Variant 2.3**	7	13	99.5	2
**Variant 2.3.1**	1	17	99.3	3
**Variant 2.3.2**	1	19	99.3	5
**Variant 2.4**	3	2.6	99.8	1
**Variant 3.0**	1	13	99.5	2

**Fig 3:**
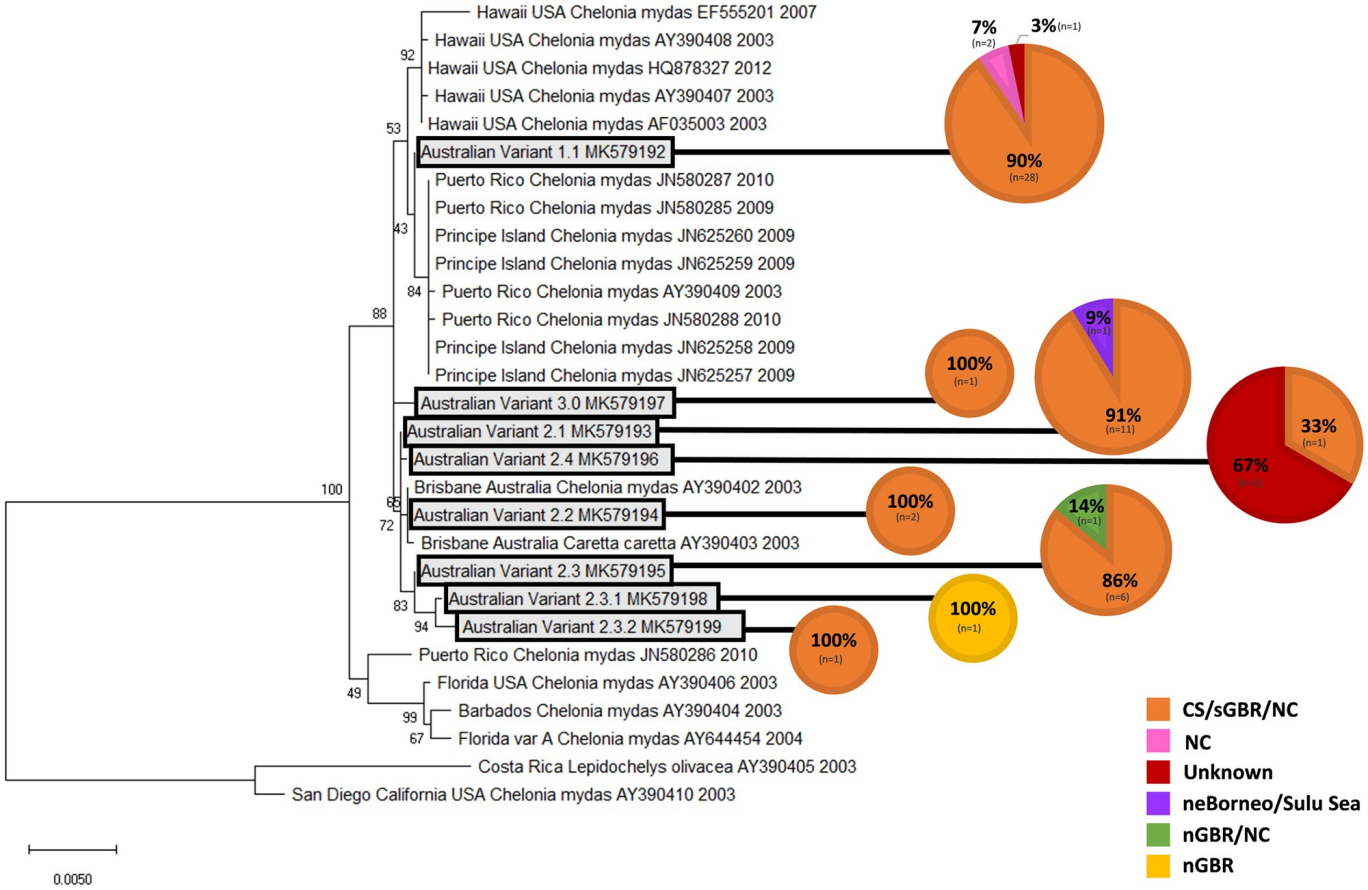
Condensed phylogenetic tree showing the positions of the distinct Australian Variants relative to published sequences. This tree was constructed using the Maximum Likelihood method generated from the aligned 2565bp ChHV5 glycoprotein B (gB) gene. The analysis involved 29 nucleotide sequences. Bootstrap values are indicated as a number on each branch and were calculated from 1000 replicates. Sequences retrieved from the GenBank database are indicated with source location, scientific name, accession number, and sample collection year. Host haplotype was used to determine the origin composition of each Australian variant in this study, expressed here as a proportion with colour reflecting host origin region; Orange = Coral Sea (CS)/southern Great Barrier Reef (sGBR)/New Caledonia (NC), Pink = NC, Red = Unknown, Purple = north-east Borneo/Sulu Sea, Green = northern Great Barrier Reef (nGBR)/NC and Yellow = nGBR.

These sequences were used to create a condensed phylogenetic tree highlighting the host haplotype origin composition of these variants (Fig 3). No apparent close relationship with turtle origin was found, as most ChHV5 variants were found in turtles from mixed origins. Only two variants were found to be from one origin only: Variant 2.2 and Variant 3.0 were both only found in samples originating from CS/sGBR/nNGR. However, both of these variants are comprised of small sample numbers (n = 2 and n = 1 respectively). Similarly, the sublineages (Variant 2.3.1 and Variant 2.3.2) were each comprised of only 1 individual, limiting conclusions as to host origins. All variants comprised of 3 or more individuals (Table 6) were isolated from individuals of mixed origins.

The results of the statistical analysis further supported that there is no association between the viral variant and the host origin (X2 = 33.771, df = 20, p = 0.290).

#### Sialyltransferase (F-sial)

**Fig 4:**
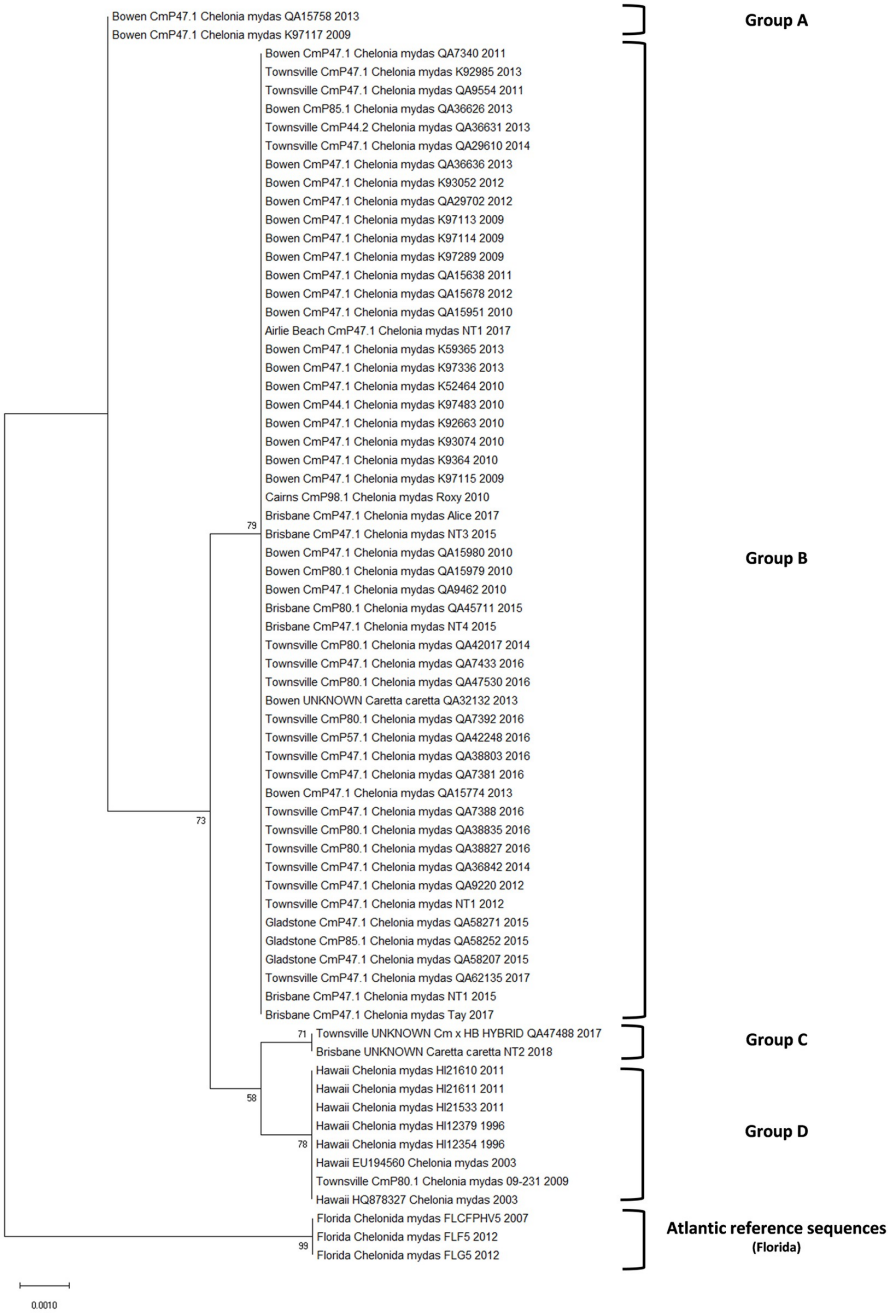
Phylogenetic tree using the Maximum Likelihood method generated from the aligned 963bp ChHV5 Sialyltransferase (F-sial) gene. The analysis involved 68 nucleotide sequences. Bootstrap values are indicated as a number on each branch and were calculated from 1000 replicates. Sequences retrieved from the GenBank database are indicated with the accession number, the source turtle’s scientific name, and sample collection location and year.

From the nucleotide and phylogenetic analysis of the 58 sequences from this study, two available full-length sequences from the NCBI database and eight published sequences [57], we show that the F-sial gene from Australian ChHV5 is highly conserved. Of the 58 sequences in this study, 52 were distinctly different from the Hawaiian reference sequences yet shared 100% similarity with each other. One sequence from Townsville (09–231) was found to be identical to the Hawaiian sequences whilst two other sequences only differed from the Hawaiian sequence by one nucleotide. Despite these minor substitutions, all sequences in the alignment shared 98.7% identical sites and this is reflected in the resulting phylogenetic tree (Fig 4). However, the highly conserved nature of these sequences indicate that this gene plays an important role in ChHV5 function.

Distinct clustering of these sequences into four main groups was observed (Fig 4). However, unlike the gB sequences generated in this study, the F-Sial sequences did not allow for viral variant characterization due to the significant similarity between these sequences. As such, these groups are named arbitrarily as Group A, B, C and D.

Group A (Accession number: MK579200) consisted of two samples which were both obtained from tumours on green turtles in Bowen and is most similar to samples collected from Florida. Group B (Accession number: MK579201) was comprised by the majority of samples (91.3%) in this study from a mixture of all study sites. All samples in this group share 100% identity. Group C (Accession number: MK579202) is comprised of two samples, one from a loggerhead and one from a green/hawksbill hybrid, and is most closely related to samples collected from Hawaii. Group D (Accession number: MK579203) is almost exclusively comprised of samples collected from Hawaii, with the exception of one turtle from Townsville (09–231) which is identical to these Hawaiian sequences.

Although the significant similarity between the F-sial sequences prevented them from clustering in the same pattern as the gB sequences, there were some commonalities between the two phylogenetic trees. For example, the gB sequence of Australian Variant 2.4 shares a six bp deletion with sequences obtained from Hawaii. The same samples that comprise Variant 2.4 in Fig 2 cluster most closely with Hawaiian sequences in Fig 4; comprising both Group C and Group D.

As with the gB sequences, we compared the sequences of these variants with a Hawaiian reference sequence (HQ878327). Whilst all Australian sequences shared a high similarity with HQ878327 (Table 7), the sequence in Group D was the most similar as it shared 100% identity. Of the 58 sequences produced in this study, 55 had a distinct amino acid change (relative to the Hawaiian reference sequences) at position 201. This indicates that this substitution is a characteristic of Australian viral variants.

**Table 7:** Nucleotide sequence analysis of sequences obtained from FP tumour samples collected from marine turtles (n). All differences and identity percentages are calculated relative to the full-length reference sequence available from Hawaii (HQ878327).

Cluster	n	Nucleotide Substitutions	Identity (%)	Non-synonymous substitutions
Group A	2	4	99.7	2
Group B	53	3	99.7	1
Group C	2	2	99.8	0
Group D	1	0	100	0

## Discussion

This study describes improved molecular assays developed for detection of ChHV5 and subsequent phylogenetic analysis. This, combined with a comprehensive sample size of individual turtles with FP tumours from a large spread of Australian sites allowed for characterisation of Australian ChHV5 variants. This formed a platform for a thorough investigation of a link between host genetic origin and ChHV5 variant, which corroborated the probability of horizontal transmission of the virus at foraging sites.

### Improved conventional PCR assays

Previous molecular studies of ChHV5 have targeted multiple genes and because detection rate is not 100% for any assay, it has been suggested that a combination of assays should be used to increase sensitivity of detection [58]. The molecular assays developed here target the F-sial, DNA polymerase and gB genes with a higher rate of detection on an individual assay basis than previously reported and can be used to amplify and sequence complete genes with Sanger sequencing, making them suitable for both ChHV5 detection and phylogenetic studies.

While none of the assays described in this study were 100% effective in detecting ChHV5 presence alone, ChHV5 presence was confirmed in all 62 individual wild-captured marine turtles with FP tumours by a combination of the 3 assays. This variability in detection is consistent with results reported in previous studies targeting ChHV5 [15, 23, 28, 58]. Comparable rates of ChHV5 detection in FP tumour samples in other studies utilizing primary conventional PCR assays ranged from 0–100% using sample sizes of between 18–37 individual turtles [15–17, 58–60]. Here, the rate of ChHV5 detection in each of the three assays in this study is in the higher range and the number of individual turtles with FP tumours sampled is comparatively larger than other studies. It is difficult to know whether this wide variation in detection rates is due to the assay or is a feature of each population sampled. However, this variability highlights the need for a standardised ChHV5 assay, which will allow for more accurate comparisons of detection rates and resulting sequences of this globally distributed virus.

Although ChHV5 is frequently detected in FP tumour samples, the variable rate of ChHV5 detection in FP tumour samples is yet to be explained. It is possible that FP development is more complex than ChHV5 presence alone [1], and there is potential for multifactorial influences on disease manifestation; these may include environmental co-factors and/or presence of other infectious agents working alone or in synergy with ChHV5 [61].

### Viral variant characterisation

The increased sample size and geographic spread, represented by six sampling locations spanning a distance of 1380km along the Queensland coast, enabled the identification and description of five main clusters of viral sequence relative to sampling location: Queensland, north Queensland, north Australian, Bowen and Brisbane. The Brisbane and Bowen clusters have not been previously reported, although the Brisbane sequences obtained in this study cluster with published sequences from Brisbane (AY390402 and AY390403). The Queensland, north Queensland and north Australian clusters of ChHV5 viral variants are previously reported [28], and our results are consistent with what is known about these clusters. The Queensland cluster includes the most common viral variant observed in both studies, which is found at many locations along the Queensland coast, whilst the north Queensland cluster contains variants that are only found in north Queensland (Townsville and Bowen). The north Australian cluster, distinct from the north Queensland Cluster, was previously reported to be comprised of viral variants obtained from FP tumours on turtles from Townsville, Cairns and Western Australia [28]. In the present study, two samples from Townsville and one sample collected in Brisbane were found to also contain this variant of ChHV5. These results are consistent with the idea that this variant is predominantly found in locations from northern Australia, but can also be found in locations great distances away [28]. However, limited sample sizes of this particular cluster in both studies prevent a conclusive understanding of the distribution of this variant.

These results suggest that there is a close relationship between ChHV5 variant and foraging ground, further supporting the theory that turtles are infected at foraging grounds, rather than rookery [2, 23, 24, 26, 28]. However, these results also indicate that viral variant distribution is not strictly homogenous at each foraging ground. For example, turtles from Bowen were found to be infected with one of three viral variants. This is consistent with ChHV5 variant distribution in foraging grounds in Florida, where multiple variants were detected within site but the frequency of each variant differed between sites [26]. Here, we also report one variant that is common amongst almost all study sites and observed most frequently within the study. Such a trend has also been reported in Florida (Variant A) [26] and Brazil (Variant 4) [23] and may reflect turtle migration patterns. Whilst turtles typically remain in a foraging ground following recruitment, small-scale movements and seasonal shifts in foraging areas have been recorded on the Queensland coast [62]. These movements could allow for exposure to other viral variants, and may explain why ChHV5 is not strictly homogenous at each location.

Prior to this study, Australian variants were referred to as “clusters” based on geographic location [28], while other studies utilised letters to denote different variants [23, 26]. Lettering systems preclude classification of sublineages, and are often unable to indicate similarity while numerical systems recognize similarity between variants and sublineages. Here, variant nomenclature was determined based on clade position in the gB phylogenetic tree, in a similar fashion to the hierarchical system used for avian influenza virus [63–65]. This classification system allowed for clear identification of Australian ChHV5 variants and sublineages; for example, Variant 2.1 and Variant 2.2 are closely related and Variant 2.3.1 is a sublineage of Variant 2.3. However, a reclassification of all known ChHV5 variants was unable to be undertaken in this study due significant variation in published gene selection and sequence length. Past variants have been determined using a partial, or complete, sequences from a range of genes. A systematic reclassification of ChHV5 using one complete gene, similar to that undertaken for Newcastle disease virus [66], would remove any ambiguities in the current phylogeny of this virus. We recommend ChHV5 gB as it is useful in both broad and small-scale phylogenic analyses. A numerical numbering system was not applied to F-sial as the highly conserved nature of the gene prevented fine-scale variant characterization, but this may change as research in this field progresses.

The F-sial gene is atypical of herpesviruses and poorly understood, but has been suggested to play a role in ChHV5 pathogenesis [49]. In this study, the F-sial gene was found to be highly conserved, with 89.6% of sequences obtained sharing 100% identity. Although this high level of similarity between sequences did not allow for fine-scale separation of variants, it indicates that this gene is highly conserved. Thus, these results are consistent with the [49] theory that F-Sial may have an important role in pathogenesis.

### Host origin and viral variant

Green turtle haplotype frequencies at rookeries are utilized by bioinformatic tools to estimate which genetic stock a particular haplotype belongs to [32, 50, 52]. Turtles frequenting a given foraging site usually represent genetic stock from multiple rookeries, although there is a trend for southern GBR haplotypes to occur at higher frequency in the more southern foraging sites and vice versa for the northern GBR stock [50, 52]. If ChHV5 were transmitted vertically from parent to offspring, then a viral variant distribution along the coast could be a reflection of turtle genetic stock distribution and phylogenetic clustering of ChHV5 would be expected to be based on host haplotype rather than sampling location.

This study used molecular methods to assess the relationship between turtle origin and viral variant, yet no close association could be identified. These results lend weight to the theory of horizontal transmission of this virus at foraging sites, rather than vertical transmission at rookeries. Fig 2 shows that the phylogenetic clustering in this study was strongly linked to sampling location, whilst Fig 3 demonstrates that each variant found in this study was isolated from turtles with a mixture of origins. However, definitive conclusions are limited as many haplotypes have been linked to multiple source regions. Most turtles in this study (76.3%) were found to belong to the CmP47.1 haplotype. This is the most common haplotype found on the GBR and has been observed in rookeries in the southern GBR, Coral Sea and New Caledonia. At present, researchers are unable to decipher which one of these three regions an individual turtle may have originated from using molecular methods. Therefore, it not yet possible to know whether all of the CmP47.1 turtles originated exclusively from the southern GBR, Coral Sea or New Caledonia, or a mixture of these regions. It has been suggested that increasing the length of mtDNA targets may allow for further differentiation of known haplotypes and more reliable identification of the region of origin for particular haplotypes [50]. The use of full mitochondrial genomic sequence, microsatellite markers, or genotype by sequencing to determine turtle haplotypes should be investigated in future studies. Despite the current limitations in establishing turtle origin by haplotype alone, the results of this study demonstrate that there is no close link between haplotype and viral variant.

### Limitations

Research on FP and ChHV5 as a causative agent is challenging as it relies on opportunistic sampling of turtles with FP tumours and thus, sample sizes are often limited. While this study has used the largest number of individual FP affected turtles to date, for some locations the sample size is still small. The limited sample availability for particular locations, coupled with some variants only being identified in a small number of turtles, prevented the assumptions of the chi-square test from being met in the statistical analysis. To address this, we reported the values from the Fisher’s exact test. However, it is clear that sampling more extensively along the GBR would greatly improve our ability to analyse and understand this disease.

This study was also somewhat limited by some inconsistency between bioinformatic programs. Australian Variant 2.4 shares a six-base deletion with published Hawaiian sequences and nucleotide analysis highlights that this variant is most closely related to these Hawaiian sequences. However, this was not accounted for in the resulting phylogenetic tree (Fig 2), despite selecting for the use of all sites in the alignment. A range of phylogenetic trees were constructed, including Neighbour-Joining, Minimum Evolution, Maximum Likelihood and Bayesian trees. Yet none of these trees reflected the similarity between these sequences, despite this deletion being repeatedly observed. This highlights limitations in some algorithms used by these programs, wherein deletions are treated as gaps and are ignored by the analysis. Such deletions may be biologically important, and a means of ensuring their inclusion in phylogenetic analysis should be targeted. However, while its position in the gB phylogenetic tree is slightly inaccurate, nucleotide analysis of Australian Variant 2.4 confirms that it is a unique and distinct group of sequences.

### Future directions

As a whole, there are still many gaps in our understanding in the biology ChHV5 and is relationship to FP. Future research on ChHV5 should aim to better understand the functional consequences of the variation observed in ChHV5 sequences. Investigations linking viral variant to disease presentation or severity would be interesting, yet challenging due to the complex nature of the disease and possible differing timelines; turtles might be captured in the early or late stage of disease development and therefore observations might be due to disease progression rather than viral variant. However, identification of a genetic link to ChHV5 pathogenicity and/or FP presentation may be possible. This disease presents differently around the world; turtles with buccal tumours are common in Hawaii yet buccal tumours have rarely been observed in Australian turtles [39]. This cause for this may be due to genetic variation of ChHV5 and should be investigated in future studies. Additionally, the results presented here, coupled with those of previous studies [49], suggest that F-Sial may play a strong role in ChHV5 pathogenicity and as such, it is worthy candidate for further investigation.

## Conclusions

While discomfort and risk to survival for individual turtles affected by FP is widely accepted, the effects of this disease on populations is less clear. Spatial and temporal variation in disease prevalence is consistently reported [1], yet a mechanism behind such variation has not been determined. The unpredictable nature of FP prevalence has so far precluded effective management plans, and researchers must endeavor to understand this disease and its associated etiological agent(s) in order to effectively conserve this vulnerable species. Here, we present a molecular epidemiological study which supports the theory that ChHV5 transmission occurs at marine turtle foraging grounds, with no close relationship to host origin. These results enable informed management decisions regarding marine turtles, as they highlight that managing FP along the Queensland coast, including the GBR, requires focus on foraging grounds.

## Supporting information

S1 Table The origin of samples used in this study, including location, turtle tag number, curved carapace length (CCL), weight and sample collection year. Whether the sample was collected from a live turtle, or during a necropsy and/or donated (^d^) is also noted. Polymerase Chain Reaction (PCR) results where the presence (+) or absence (−) of chelonid alphaherpesvirus 5 (ChHV5) in FP tumour samples collected from turtles with different capture locations and host haplotype is also reported. All samples were collected from green turtles, excluding two sample from loggerheads (*) and one from a green/hawksbill hybrid (**).
(DOCX)

## References

[1] Jones K, Ariel E, Burgess G, Read M. A review of fibropapillomatosis in Green turtles (Chelonia mydas). The Veterinary Journal. 2016;212:48–57. 10.1016/j.tvjl.2015.10.041 27256025

[2] Herbst LH. Fibropapillomatosis of marine turtles. Annual Review of Fish Diseases. 1994;4:389–425. 10.1016/0959-8030(94)90037-X

[3] Flint M, Limpus CJ, Patterson-Kane JC, Murray PJ, Mills PC. Corneal Fibropapillomatosis in Green Sea Turtles (Chelonia mydas) in Australia. Journal of Comparative Pathology. 2010;142(4):341–6. 10.1016/j.jcpa.2009.10.012 19954789

[4] Work TM, Balazs GH, Rameyer RA, Morris RA. Retrospective pathology survey of green turtles Chelonia mydas with fibropapillomatosis in the Hawaiian Islands, 1993–2003. Diseases of aquatic organisms. 2004;62(1–2):163–76. 10.3354/dao062163 15648843

[5] Aguirre AA, Balazs GH, Spraker TR, Gross TS. Adrenal and Hematological Responses to Stress in Juvenile Green Turtles (Chelonia mydas) with and without Fibropapillomas. Physiological Zoology. 1995;68(5):831–54.

[6] Work TM, Rameyer RA, Balazs GH, Cray C, Chang SP. Immune status of free-ranging green turtles with fibropapillomatosis from Hawaii. Journal of wildlife diseases. 2001;37(3):574 10.7589/0090-3558-37.3.574 11504232

[7] Jacobson ER, Buergelt C, Williams B, Harris RK. Herpesvirus in cutaneous fibropapillomas of the green turtle Chelonia mydas. Diseases of Aquatic Organisms. 1991;12:1–6. 10.3354/dao012001

[8] Jacobson ER, Mansell JL, Sundberg JP, Hajjar L, Reichmann ME, Ehrhart LM, et al Cutaneous fibropapillomas of green turtles (*Chelonia mydas*). Journal of Comparative Pathology. 1989;101(1):39–52. 10.1016/0021-9975(89)90075-3 2677067

[9] Herbst LH, Jacobson ER, Moretti R, Brown T, Sundberg JP, Klein PA. Experimental transmission of green turtle fibropapillomatosis using cell-free tumor extracts. Diseases of Aquatic Organisms. 1995;22:1–12. 10.3354/dao022001

[10] Work TM, Dagenais J, Weatherby TM, Balazs GH, Ackermannd M. In vitro replication of chelonid herpesvirus 5 in organotypic skin cultures from Hawaiian green turtles (Chelonia mydas). Journal of Virology. 2017;91(17). 10.1128/JVI.00404-17 28615209 PMC5553171

[11] Lackovich JK, Jacobson ER, Curry SS, Klein PA, Brown DR, Homer BL, et al Association of herpesvirus with fibropapillomatosis of the green turtle *Chelonia mydas* and the loggerhead turtle *Caretta caretta* in Florida. Diseases of aquatic organisms. 1999;37(2):89–97. 10.3354/dao037089 10494499

[12] Lu Y, Wang Y, Yu Q, Aguirre AA, Balazs GH, Nerurkar VR, et al Detection of herpesviral sequences in tissues of green turtles with fibropapilloma by polymerase chain reaction. Archives of virology. 2000;145(9):1885–93. 10.1007/s007050070063 11043948

[13] Lu YA, Wang Y, Aguirre AA, Zhao ZS, Liu CY, Nerurkar VR, et al RT-PCR detection of the expression of the polymerase gene of a novel reptilian herpesvirus in tumor tissues of green turtles with fibropapilloma. Archives of virology. 2003;148(6):1155–63. 10.1007/s00705-002-0970-8 12756620

[14] Page-Karjian A, Norton TM, Ritchie B, Brown C, Mancia C, Jackwood M, et al Quantifying chelonid herpesvirus 5 in symptomatic and asymptomatic rehabilitating green sea turtles. Endangered Species Research. 2015;28(2):135–46. 10.3354/esr00687

[15] Page-Karjian A, Torres F, Zhang J, Rivera S, Diez C, Moore PA, et al Presence of chelonid fibropapilloma-associated herpesvirus in tumored and non-tumored green turtles, as detected by polymerase chain reaction, in endemic and non-endemic aggregations, Puerto Rico. SpringerPlus. 2012;1(1):1–8. 10.1186/2193-1801-1-35 23961364 PMC3725908

[16] Quackenbush SL, Aguirre AA, Spraker TR, Horrocks JA, Vermeer LA, Balazs GH, et al Quantitative analysis of herpesvirus sequences from normal tissue and fibropapillomas of marine turtles with real-time PCR. Virology. 2001;287(1):105–11. 10.1006/viro.2001.1023 11504546

[17] Quackenbush SL, Bowser PR, Work TM, Balazs GH, Casey RN, Casey JW, et al Three Closely Related Herpesviruses Are Associated with Fibropapillomatosis in Marine Turtles. Virology. 1998;246(2):392–9. 10.1006/viro.1998.9207 9657957

[18] Nigro O, Alonso Aguirre A, Lu Y. Nucleotide sequence of an ICP18.5 assembly protein (UL28) gene of green turtle herpesvirus pathogenically associated with green turtle fibropapilloma. Journal of virological methods. 2004;120(1):107–12. 10.1016/j.jviromet.2004.04.011 15234815

[19] Nigro O, Yu G, Aguirre AA, Lu Y. Sequencing and characterization of the full-length gene encoding the single-stranded DNA binding protein of a novel Chelonian herpesvirus. Archives of virology. 2004;149(2):337–47. 10.1007/s00705-003-0204-8 14745599

[20] Yu Q, Hu N, Lu Y, Nerurkar VR, Yanagihara R. Rapid acquisition of entire DNA polymerase gene of a novel herpesvirus from green turtle fibropapilloma by a genomic walking technique. Journal of virological methods. 2001;91(2):183–95. 10.1016/s0166-0934(00)00267-6 11164500

[21] Yu Q, Lu Y, Nerurkar VR, Yanagihara R. Amplification and analysis of DNA flanking known sequences of a novel herpesvirus from green turtles with fibropapilloma Brief report. Archives of virology. 2000;145(12):2669 10.1007/s007050070015 11205112

[22] Alfaro-Nunez A, Bertelsen MF, Bojesen AM, Rasmussen I, Zepeda-Mendoza L, Olsen MT, et al Global distribution of Chelonid fibropapilloma-associated herpesvirus among clinically healthy sea turtles. BMC Evolutionary Biology. 2014;14(1). 10.1186/s12862-014-0206-z 25342462 PMC4219010

[23] Rodenbusch CR, Baptistotte C, Werneck MR, Pires TT, Melo MTD, de Ataíde MW, et al Fibropapillomatosis in green turtles Chelonia mydas in Brazil: characteristics of tumors and virus. Diseases of aquatic organisms. 2014;111(3):207–17. 10.3354/dao02782 25320033

[24] Patrício AR, Herbst LH, Duarte A, Vélez-Zuazo X, Santos Loureiro N, Pereira N, et al Global phylogeography and evolution of chelonid fibropapilloma-associated herpesvirus. Journal of general virology. 2012;93:1035 10.1099/vir.0.038950-0 22258862

[25] Cardenas DM, Cucalon RV, Medina-Magues LG, Jones K, Aleman RA, Alfaro-Nunez A, et al Fibropapillomatosis in a Green Sea Turtle (Chelonia mydas) from the Southeastern Pacific. J Wildl Dis. 2018;In-Press. Epub 2018/08/11. 10.7589/2017-12-295 . 30096036

[26] Ene A, Su M, Lemaire S, Rose C, Schaff S, Moretti R, et al Distribution of chelonid fibropapillomatosis-associated herpesvirus variants in Florida: molecular genetic evidence for infection of turtles following recruitment to neritic developmental habitats. Journal of wildlife diseases. 2005;41(3):489 10.7589/0090-3558-41.3.489 16244058

[27] Herbst L, Ene A, Su M, Desalle R, Lenz J. Tumor outbreaks in marine turtles are not due to recent herpesvirus mutations. Current Biology. 2004;14(17):R697–R9. 10.1016/j.cub.2004.08.040 15341757

[28] Ariel E, Nainu F, Jones K, Juntunen K, Bell I, Gaston J, et al Phylogenetic Variation of Chelonid Alphaherpesvirus 5 (ChHV5) in Populations of Green Turtles Chelonia mydas along the Queensland Coast, Australia. Journal of Aquatic Animal Health. 2017;29(3):150–7. 10.1080/08997659.2017.1330783 28524816

[29] Greenblatt RJ, Balazs GH, Casey JW, Work TM, Dutton P, Sutton CA, et al Geographic variation in marine turtle fibropapillomatosis. Journal of Zoo and Wildlife Medicine. 2005;36(3):527–30. 10.1638/04-051.1 17312778

[30] Reich KJ, Bjorndal KA, Bolten AB. The ‘lost years’ of green turtles: using stable isotopes to study cryptic lifestages. Biology Letters. 2007;3(6):712–4. 10.1098/rsbl.2007.0394 17878144 PMC2391226

[31] Anderson JD, Shaver DJ, Karel WJ. Genetic Diversity and Natal Origins of Green Turtles (Chelonia mydas) in the Western Gulf of Mexico. Journal of Herpetology. 2013;47(2):251–7. 10.1670/12-031

[32] Dutton PH, Jensen MP, Frutchey K, Frey A, LaCasella E, Balazs GH, et al Genetic Stock Structure of Green Turtle (Chelonia mydas) Nesting Populations Across the Pacific Islands. Pacific Science. 2014;68(4):451–64. 10.2984/68.4.1

[33] Lahanas PN, Bjorndal KA, Bolten AB, Encalada SE, Miyamoto MM, Valverde RA, et al Genetic composition of a green turtle (Chelonia mydas) feeding ground population: evidence for multiple origins. Marine Biology. 1998;130(3):345–52. 10.1007/s002270050254

[34] Musick JA, Limpus C. Habitat utilization and migration in juvenile sea turtles In: Lutz PL, Musick JA, editors. The biology of sea turtles. 1 United States of America: CRC Press; 1997 p. 137–63.

[35] Limpus CJ. A biological review of Australian marine turtle species 2 Green turtle, *Chelonia mydas* (Linnaeus). Queensland, Australia: Queensland Environmental Protection Agency, 2008.

[36] Chaloupka M, Bjorndal KA, Balazs GH, Bolten AB, Ehrhart LM, Limpus CJ, et al Encouraging outlook for recovery of a once severely exploited marine megaherbivore. Global Ecology and Biogeography. 2008;17(2):297–304. 10.1111/j.1466-8238.2007.00367.x

[37] Dobbs K. MARINE TURTLES in the Great Barrier Reef World Heritage Area. Queensland: 2001.

[38] Gulko D, and Eckert K. Sea turtles: An Ecological Guide. Hawaii: Mutual Publishing; 2004.

[39] Hargrove S, Work T, Brunson S, Foley AM, Balazs G. Proceedings of the 2015 international summit on fibropapillomatosis: global status, trends, and population impacts. NOAA Technical Memorandum. 2016;NOAA-TM-NMFS-PIFSC-54,:87.

[40] Limpus CJ, Reed PC. The green turtle, *Chelonia mydas*, in Queensland, a preliminary description of the population structure in a coral reef feeding ground In: Grigg GC, Shine R, Ehmann H, editors. Biology of Australasian frogs and reptiles. Chipping Norton, N.S.W: Surrey Beatty in association with The Royal Zoological Society of New South Wales; 1985 p. 47–52.

[41] Limpus CJ, Couper PJ, Read MA. The loggerhead turtle, Caretta caretta, in Queensland: Population structure in a warm temperate feeding area. Memoirs of the Queensland Museum Brisbane. 1994;37(1):195–204.

[42] Limpus CJ. The hawksbill turtle, Eretmochelys imbricata, in Queensland: population structure within a southern Great Barrier Reef feeding ground. Wildlife Research. 1992;19(4):489–505. 10.1071/WR9920489

[43] Limpus CJ, Couper PJ, Read MA. The green turtle, Chelonia mydas, in Queensland: Population structure in a warm temperature feeding area. Memoirs of the Queensland Museum Brisbane. 1994;35(1):139–54.

[44] Lu Y, Yu Q, Zamzow JP, Wang Y, Losey GS, Balazs GH, et al Detection of Green Turtle Herpesviral Sequence in Saddleback WrasseThalassoma duperrey: A Possible Mode of Transmission of Green Turtle Fibropapilloma. Journal of Aquatic Animal Health. 2000;12(1):58–63. 10.1577/1548-8667(2000)012<0058:DOGTHS>2.0.CO;2 28880781

[45] Monezi TA, Mehnert DU, de Moura EMM, Müller NMG, Garrafa P, Matushima ER, et al Chelonid herpesvirus 5 in secretions and tumor tissues from green turtles (Chelonia mydas) from Southeastern Brazil: A ten-year study. Veterinary microbiology. 2016;186:150–6. 10.1016/j.vetmic.2016.02.020 27016769

[46] Origgi FC, Tecilla M, Pilo P, Aloisio F, Otten P, Aguilar-Bultet L, et al A Genomic Approach to Unravel Host-Pathogen Interaction in Chelonians: The Example of Testudinid Herpesvirus 3. PLoS One. 2015;10(8):e0134897 Epub 2015/08/06. 10.1371/journal.pone.0134897 . 26244892 PMC4526542

[47] Bender FC, Samanta M, Heldwein EE, Manuel Ponce de L, Bilman E, Lou H, et al Antigenic and Mutational Analyses of Herpes Simplex Virus Glycoprotein B Reveal Four Functional Regions. Journal of Virology. 2007;81(8):3827–41. 10.1128/JVI.02710-06 17267495 PMC1866100

[48] Coberley SS, Condit RC, Herbst LH, Klein PA. Identification and Expression of Immunogenic Proteins of a Disease-Associated Marine Turtle Herpesvirus. Journal of Virology. 2002;76(20):10553–8. 10.1128/JVI.76.20.10553-10558.2002 12239336 PMC136575

[49] Ackermann M, Leong J-AC, Koriabine M, Hartmann-Fritsch F, de Jong PJ, Lewis TD, et al The genome of Chelonid herpesvirus 5 harbors atypical genes. Public Library of Science. 2012;7(10):e46623. 10.1371/journal.pone.0046623 PMC3462797 23056373

[50] Jones K, Jensen M, Burgess G, Leonhardt J, van Herwerden L, Hazel J, et al Closing the gap: Mixed stock analysis of three foraging populations of green turtles (*Chelonia mydas*) on the Great Barrier Reef. PeerJ. 2018;6(e5651). 10.7717/peerj.5651. PMC6166616 30280029

[51] Kearse M, Moir R, Wilson A, Stones-Havas S, Cheung M, Sturrock S, et al Geneious Basic: an integrated and extendable desktop software platform for the organization and analysis of sequence data. Bioinformatics (Oxford, England). 2012;28(12):1647–9. Epub 2012/05/01. 10.1093/bioinformatics/bts199 . 22543367 PMC3371832

[52] Jensen MP, Bell I, Limpus CJ, Hamann M, Ambar S, Whap T, et al Spatial and temporal genetic variation among size classes of green turtles (Chelonia mydas) provides information on oceanic dispersal and population dynamics. Marine Ecology Progress Series. 2016;543:241–56.

[53] Thompson JD, Thompson JD, Higgins DG, Higgins DG, Gibson TJ, Gibson TJ. CLUSTAL W: Improving the sensitivity of progressive multiple sequence alignment through sequence weighting, position-specific gap penalties and weight matrix choice. Nucleic Acids Research. 1994;22(22):4673–80. 10.1093/nar/22.22.4673 7984417 PMC308517

[54] Kumar S, Stecher G, Li M, Knyaz C, Tamura K. MEGA X: Molecular Evolutionary Genetics Analysis across Computing Platforms. MOLECULAR BIOLOGY AND EVOLUTION. 2018;35(6):1547–9. 29722887 10.1093/molbev/msy096 PMC5967553

[55] Kimura M. A simple method for estimating evolutionary rates of base substitutions through comparative studies of nucleotide sequences. Journal of molecular evolution. 1980;16(2):111–20. 10.1007/bf01731581 7463489

[56] Jukes TH, Cantor CR. Evolution of protein molecules In: Munro HN, editor. Mammalian Protein Metabolism. New Yo: Academic Press; 1969 p. 21–132.

[57] Morrison CL, Iwanowici L, Work TM, Fahsbender E, Breitbart M, Adams C, et al Genomic evolution, recombination, and inter-strain diversity of chelonid alphaherpesvirus 5 from Florida and Hawaii green sea turtles with fibropapillomatosis. PeerJ. 2018;6(2):e4386 10.7717/peerj.4386 29479497 PMC5824677

[58] Alfaro-Núñez A, Gilbert TP. Validation of a sensitive PCR assay for the detection of Chelonid fibropapilloma-associated herpesvirus in latent turtle infections. Journal of virological methods. 2014;206:38–41. 10.1016/j.jviromet.2014.05.019 24882497 PMC7119791

[59] Ariel E, Lee K, Jones K, Scott J, Picard J. Arcobacter infection and temporary regression of tumours in a green turtle (*Chelonia mydas*) with fibropapillomatosis. Australian Veterinary Journal. 2017;Submitted.

[60] Lawrance MF, Mansfield KL, Sutton E, Savage AE. Molecular evolution of fibropapilloma-associated herpesviruses infecting juvenile green and loggerhead sea turtles. Virology. 2018;521:190–7. 10.1016/j.virol.2018.06.012 29960922

[61] Mashkour N, Maclaine A, Burgess GW, Ariel E. Discovery of an Australian Chelonia mydas papillomavirus via green turtle primary cell culture and qPCR. Journal of Virological Methods. 2018;258:13–23. 10.1016/j.jviromet.2018.04.004 29630942

[62] Shimada T, Jones R, Limpus C, Groom R, Hamann M. Long-term and seasonal patterns of sea turtle home ranges in warm coastal foraging habitats: Implications for conservation. Marine Ecology Progress Series. 2016;562:163–79. 10.3354/meps11972

[63] Brown IH, Capua I, Cattoli G, Chen HL, Cox N, Davis CT, et al Continuing progress towards a unified nomenclature for the highly pathogenic H5N1 avian influenza viruses: divergence of clade 2.2 viruses. INFLUENZA AND OTHER RESPIRATORY VIRUSES. 2009;3(2):59–62. 10.1111/j.1750-2659.2009.00078.x 19496842 PMC4634523

[64] Donis RO, Smith GJD, Perdue ML, Brown IH, Chen H, Fouchier RAM, et al Toward a unified nomenclature system for highly pathogenic avian influenza virus (H5N1). Emerging Infectious Diseases. 2008;14(7):e1–e. 10.3201/eid1407.071681 18598616 PMC2600337

[65] Smith GJD, Donis RO, Working WOFHNE, Group WOFHNEW. Continued evolution of highly pathogenic avian influenza A (H5N1): updated nomenclature. Influenza and Other Respiratory Viruses. 2012;6(1):1–5. 10.1111/j.1750-2659.2011.00298.x 22035148 PMC5074649

[66] Diel DG, da Silva LHA, Liu H, Wang Z, Miller PJ, Afonso CL. Genetic diversity of avian paramyxovirus type 1: Proposal for a unified nomenclature and classification system of Newcastle disease virus genotypes. Infection, Genetics and Evolution. 2012;12(8):1770–9. 10.1016/j.meegid.2012.07.012 22892200

