# Supplementary material for: Molecular evidence for horizontal transmission of chelonid alphaherpesvirus 5 at green turtle (Chelonia mydas) foraging grounds in Queensland, Australia
Source: PLoS One. 2020 Jan 9;15(1):e0227268. doi: 10.1371/journal.pone.0227268 (PMC6952114; doi:10.1371/journal.pone.0227268)
Supplement: S1 Table — Whether the sample was collected from a live turtle, or during a necropsy and/or donated (d) is also noted. Polymerase Chain Reaction (PCR) results where the presence (+) or absence (−) of chelonid alphaherpesvirus 5 (ChHV5) in FP tumour samples collected from turtles with different capture locations and host haplotype is also reported. All samples were collected from green turtles, excluding two sample from loggerheads (*) and one from a green/hawksbill hybrid (**). (DOCX) [file pone.0227268.s001.docx]

| **Collection Details** | | | | | **Results** | | | |
| --- | --- | --- | --- | --- | --- | --- | --- | --- |
| **Location** | **Tag No.** | **CCL** | **Sample Year** | **Collection Type** | **Host Haplotype** | **DNApol** | **gB** | **Fsial** |
| Cairns | Roxy | 47.6 | 2010 | Live turtle | CmP98.1 | + | + | + |
| Townsville | QA42923 | 43.6 | 2016 | Live turtle | CmP34.1 | + | **-** | **-** |
| Townsville | QA36631 | 59.6 | 2013 | Live turtle | CmP44.2 | + | + | + |
| Townsville | K92985 | 46.2 | 2013 | Live turtle | CmP47.1 | + | + | + |
| Townsville | QA15682 | 49.7 | 2013 | Live turtle | CmP47.1 | + | **-** | **-** |
| Townsville | QA29610 | 45.0 | 2014 | Live turtle | CmP47.1 | + | + | + |
| Townsville | QA36842 | 46.3 | 2014 | Live turtle | CmP47.1 | + | + | + |
| Townsville | QA38803 | 50.5 | 2016 | Live turtle | CmP47.1 | + | + | + |
| Townsville | QA62135 | 48.0 | 2017 | Live turtle | CmP47.1 | **-** | + | + |
| Townsville | QA7381 | 53.0 | 2016 | Live turtle | CmP47.1 | + | + | + |
| Townsville | QA7388 | 49.5 | 2016 | Live turtle | CmP47.1 | + | + | + |
| Townsville | QA7433 | 44.9 | 2016 | Live turtle | CmP47.1 | + | + | + |
| Townsville | QA9220 | 70.2 | 2012 | Live turtle | CmP47.1 | + | + | + |
| Townsville | QA9554 | 53.7 | 2011 | Live turtle | CmP47.1 | + | + | + |
| Townsville | TSV-NT1 | Unknown | 2012 | Necropsy | CmP47.1 | + | + | + |
| Townsville | QA42248 | 44.2 | 2016 | Live turtle | CmP57.1 | + | + | + |
| Townsville | 09-231 | 57.1 | 2009 | Live turtle | CmP80.1 | + | + | + |
| Townsville | QA38827 | 44.0 | 2016 | Live turtle | CmP80.1 | + | + | + |
| Townsville | QA38835 | 50.3 | 2016 | Live turtle | CmP80.1 | + | + | + |
| Townsville | QA42017 | 48.6 | 2014 | Live turtle | CmP80.1 | + | + | + |
| Townsville | QA47530 | 48.1 | 2016 | Live turtle | CmP80.1 | + | + | + |
| Townsville | QA7392 | 50.2 | 2016 | Live turtle | CmP80.1 | + | + | + |
| Townsville | QA47488****** | 60.3 | 2017 | Necropsy | N/A | + | + | + |
| Bowen | K97483 | 48.5 | 2010 | Live turtle | CmP44.1 | + | + | + |
| Bowen | K52464 | 54.0 | 2010 | Live turtle | CmP47.1 | **-** | + | + |
| Bowen | K59365 | 46.5 | 2013 | Live turtle | CmP47.1 | + | + | + |
| Bowen | K92663 | 44.7 | 2010 | Live turtle | CmP47.1 | + | + | + |
| Bowen | K93038 | 49.6 | 2013 | Live turtle | CmP47.1 | + | + | **-** |
| Bowen | K93052 | 42.5 | 2012 | Live turtle | CmP47.1 | + | + | + |
| Bowen | K93074 | 45.0 | 2010 | Live turtle | CmP47.1 | + | + | + |
| Bowen | K93640 | 47.9 | 2010 | Live turtle | CmP47.1 | + | + | + |
| Bowen | K97113 | 47.9 | 2009 | Live turtle | CmP47.1 | + | + | + |
| Bowen | K97114 | 50.5 | 2009 | Live turtle | CmP47.1 | + | + | + |
| Bowen | K97115 | 54.3 | 2009 | Live turtle | CmP47.1 | + | + | + |
| Bowen | K97117 | 45.0 | 2009 | Live turtle | CmP47.1 | + | + | + |
| Bowen | K97289 | 51.8 | 2009 | Live turtle | CmP47.1 | **-** | + | + |
| Bowen | K97336 | 25.7 | 2013 | Live turtle | CmP47.1 | + | + | + |
| Bowen | QA15638 | 48.6 | 2011 | Live turtle | CmP47.1 | + | + | + |
| Bowen | QA15678 | 76.2 | 2012 | Live turtle | CmP47.1 | + | + | + |
| Bowen | QA15758 | 49.4 | 2013 | Live turtle | CmP47.1 | + | + | + |
| Bowen | QA15774 | 48.7 | 2013 | Live turtle | CmP47.1 | + | + | + |
| Bowen | QA15951 | 45.4 | 2010 | Live turtle | CmP47.1 | + | + | + |
| Bowen | QA15980 | 44.0 | 2010 | Live turtle | CmP47.1 | + | + | + |
| Bowen | QA29702 | 44.4 | 2012 | Live turtle | CmP47.1 | + | + | + |
| Bowen | QA36626 | 44.8 | 2013 | Live turtle | CmP47.1 | + | + | + |
| Bowen | QA7340 | 45.3 | 2011 | Live turtle | CmP47.1 | + | + | + |
| Bowen | QA9462-1 | 44.5 | 2010 | Live turtle | CmP47.1 | + | + | + |
| Bowen | QA15979 | 47.2 | 2010 | Live turtle | CmP80.1 | + | + | + |
| Bowen | QA36636 | 47.6 | 2013 | Live turtle | CmP85.1 | + | + | + |
| Bowen | QA32132***** | 85.5 | 2013 | Live turtle | N/A | + | + | + |
| Airlie Beach | AB-NT1 | 49.0 | 2017 | Necropsy^d^ | CmP47.1 | + | + | + |
| Gladstone | QA34793 | 60.2 | 2015 | Live turtle | CmP47.1 | + | + | + |
| Gladstone | QA58252 | 70.1 | 2015 | Live turtle | CmP47.1 | + | **-** | **-** |
| Gladstone | QA58271 | 70.1 | 2015 | Live turtle | CmP47.1 | **-** | **-** | + |
| Gladstone | QA58207 | 60.3 | 2015 | Live turtle | CmP85.1 | + | + | + |
| Brisbane | Alice | 45.0 | 2017 | Necropsy^d^ | CmP47.1 | + | + | + |
| Brisbane | MB-NT1 | 46.3 | 2015 | Necropsy | CmP47.1 | + | + | + |
| Brisbane | MB-NT3 | 44.4 | 2015 | Necropsy | CmP47.1 | + | + | + |
| Brisbane | MB-NT4 | 43.1 | 2015 | Necropsy | CmP47.1 | + | + | + |
| Brisbane | Tay | 52.4 | 2017 | Necropsy^d^ | CmP47.1 | + | + | + |
| Brisbane | QA45711 | 52.1 | 2015 | Live turtle | CmP80.1 | + | + | + |
| Brisbane | MB-NT2***** | 98.0 | 2018 | Necropsy^d^ | N/A | + | + | + |
|  |  |  |  | Total | 62 | 58 | 58 | 58 |
